# Pharmacokinetic profile and partitioning in red blood cells of romifidine after single intravenous administration in the horse

**DOI:** 10.1002/vms3.70

**Published:** 2017-07-20

**Authors:** Noemi Romagnoli, Khaled M. Al‐Qudah, Sara Armorini, Carlotta Lambertini, Anna Zaghini, Alessandro Spadari, Paola Roncada

**Affiliations:** ^1^ Department of Veterinary Medical Sciences (UNI EN ISO 9001:2008) University of Bologna Ozzano Emilia Italy; ^2^ Department of Veterinary Clinical Sciences Faculty of Veterinary Medicine Jordan University of Science and Technology Irbid Jordan

**Keywords:** horse, romifidine, sedation score, RBC kinetics

## Abstract

The aims of this study were to assess the plasma concentrations of romifidine in horses after intravenous injection, to evaluate the red blood cell (RBC) partitioning of the anaesthetic drug, and to improve knowledge regarding its sedative effect in horses describing the pharmacokinetic model. Eight adult Standardbred horses received a single bolus of romifidine at a dosage of 100 *μ*g/kg. Blood samples (5 mL) were collected immediately before romifidine administration (*t*_0_), and at 2, 5, 10, 15, 20, 25, 30, 40, 50, 60, 75, 90, 105, 120, 150 and 180 min after injection. A sedation score was recorded at the same time. The romifidine concentrations in plasma and red blood cells were determined by high performance liquid chromatography (HPLC). The plasma and red blood cell concentrations were correlated with the sedation at each time point. Romifidine produced a satisfactory level of sedation in all animals. The sedation was detectable in all horses for up to 105 min. All the animals returned to normal without any behavioural changes at 180 min. The romifidine concentrations in the red blood cells were significantly higher (*P* < 0.01) at all time points than those in the plasma. The T_1/2*β*_ was 148.67 ± 61.59 min and body clearance was 22.55 ± 6.67 mL/kg per min. The results showed that after a single bolus administration of romifidine, a partitioning in the RBCs was detected.

## Introduction

The use of alpha‐2 adrenoceptor agonist drugs has gained wide acceptance in equine veterinary practice (Marzok *et al*. 2014). Xylazine, detomidine and romifidine have currently been approved for use in Europe (Reg. CE N. 1950/2006) and are used for purposes of sedation, analgesia and premedication (England & Clarke 1996; Gozalo‐Marcilla *et al*. 2015).

Romifidine [N‐(2‐bromo‐6‐fluoro‐phenyl)‐4,5‐dihydro‐1H‐imidazol‐2‐amine mono‐hydrochloride, C_9_H_9_BrFN_3_], is commonly administered intravenously to produce sedation and provide analgesia in horses. The sedative effects reported are decreased awareness, ptosis of the head, lower lip and eyelids, ataxia and a wide stance. Similar to other alpha‐2 agonists, the side effects related to the administration of romifidine are bradycardia, arrhythmias, decreased cardiac output and increased systemic vascular resistance, respiratory depression, transient decreases in arterial partial pressure of oxygen and ataxia (Wojtasiak‐Wypart *et al*. 2012; Gozalo‐Marcilla *et al*. 2015). When compared with IV xylazine (1 mg/kg) or detomidine (20 *μ*g/kg), romifidine at 40 and 80 *μ*g/kg produced less ataxia and the horses’ heads were held higher; furthermore, the duration of these effects was longer than the others. The lack of ataxia with romifidine has made it very popular for sedating horses in standing procedures, and it is also widely used for premedication prior to induction of anaesthesia in this species (Marzok *et al*. 2014).

Romifidine in horses is usually administered intravenously (IV) at a dosage ranging from 40 to120 *μ*g/kg (Freeman *et al*. 2002; Figueiredo *et al*. 2005). Several pharmacodynamic/pharmacokinetic studies have shown a prolonged sedative effect at the higher doses and a phase of residual sedation, detectable for 10 h after drug administration (Poulsen Nautrup 1988; England *et al*. 1992; Wojtasiak‐Wypart *et al*. 2012).

Romifidine is a lipophilic drug and easily crosses cell membranes by passive diffusion. In human medicine, several *in vitro* studies have suggested the ability of lipophilic drugs to pass the membranes of red blood cells (RBCs) (Hinderling 1984, 1997; Reichel *et al*. 1994). Among the cellular constituents of blood, RBCs represent the largest population both in number and cell size, and some authors have suggested their important role in pharmacokinetic/pharmacodynamic behaviour (Hinderling 1997; Loos *et al*. 2003). Pharmacokinetic parameters are usually determined by analysis of drug concentrations in plasma rather than whole blood. Parameters determined using plasma data may be misleading if concentrations of drug differ between plasma and RBCs (Caglar *et al*. 2015). Red blood cells may unintentionally serve as a natural blood compartment participating in biodistribution, pharmacokinetics, slow release, metabolism and action of different drugs (Muzykantov 2010).

Previous studies have described the pharmacokinetics of ketamine in plasma and in RBCs in cats, dogs and horses, explaining the great variability in the response of these species to the same drug dosage (Roncada *et al*. 2003, 2005; Sori *et al*. 2013) but, to the best of our knowledge, no data have been reported in horses regarding the concentrations of romifidine in RBCs after intravenous administration. The aims of the present study were to assess the plasma concentrations of romifidine in horses after intravenous injection and to compare these concentrations with those found in RBCs in order to evaluate the RBC partitioning of the alpha‐2 adrenoceptor drug.

## Materials and Methods

### Animals

Eight client‐owned Standardbred horses (four males and four females) 8.5 ± 3.6 (mean value ± SD) years old, weighing 402 ± 32.7 kg admitted to the Veterinary Teaching Hospital for diagnostic procedure, were enrolled in the study. The owners of the animals were informed of the purpose of the study. All owners provided written informed consent prior to enrolment of the horses. The study was approved by the Scientific Ethics Committee for Experimentation on Animals of Alma Mater Studiorum‐University of Bologna. The care and handling of the animals were in accordance with the provisions of European Economic Community Council Directive 86/609, adopted by the Italian Government (D.L. 27/01/1992 no. 116).

### The study was carried out in September and October 2012

The animals were housed in stalls of the Veterinary Teaching Hospital of the Department of Veterinary Medical Sciences for a 3 days period of acclimatization. The animals were fasted for twelve hours before the moment of sedation but had free access to drinking water. According to the classification of the American Society of Anaesthesiology (ASA), based on their history, physical examination and clinicopathological evaluation, (the horses were classified as ASA I). The range of haematocrit value was 35–38% (normal range 32–52%).

Three hours prior to the experiment, the horses were prepared for intravenous injection by shaving the hair and washing the skin over the left and the right jugular veins with surgical soap. The areas were then infiltrated with 1 mL 2% lidocaine (ATI, Bologna, Italy). In all horses, a 14‐gauge catheter (Milacath, Mila International, USA) was placed into the right jugular vein for the collection of blood samples, and a 16‐gauge catheter (Milacath) was placed into the left jugular vein for drug administration The catheter were sutured with silk suture material. Each horse remained undisturbed in individual boxes, and romifidine (Sedivet^®^ 1%; Boheringer Ingelheim Vetmedica, Milan, Italy) was administered intravenously in a single bolus to the horses at a dosage of 100 *μ*g/kg.

### Blood sample collection

The catheter in the right jugular vein was used for blood sampling. Blood samples (5 mL) were collected immediately before romifidine administration (*t*_0_), and at 2, 5, 10, 15, 20, 25, 30, 40, 50, 60, 75, 90, 105, 120, 150 and 180 min after injection. All blood samples were immediately transferred to heparinised tubes and centrifuged (1200*g*) for 20 min at room temperature. After centrifugation, the plasma was separated from the entire cellular fraction containing RBCs; both biological matrices were transferred into separated clean test tubes and frozen at −20°C until analysis (about 4 weeks).

### Sedation score

Before each blood sampling, each horse underwent measurement of the degree of sedation using a modified score proposed by Figueiredo *et al*. (2005) and summarized in Table 1. All evaluations were carried out by the same expert anaesthetist (NR).

**Table 1:** Description of sedation score

score	Behaviour
0	No sedation (normal frequency and velocity of movement, ear and neck carriage, eye alertness, lip apposition, postural tone, stance), the head level above the wither
1	Mild sedation (slightly decreased frequency and velocity of movement, lower ear and neck carriage, reduced eye alertness, appearance of lip separation, slight base‐wide stance, slightly relaxed postural tone), the head level at the same level as the wither
2	Moderate sedation (moderately decreased frequency and velocity of movement, obvious ear tip separation, increased base‐wide stance, appearance of crossed legs, buckled knees and/or fetlocks, more relaxed postural tone), the head level lower than the wither (at the shoulder one)
3	Deep sedation (markedly decreased frequency and velocity of movement, pronounced ear tip separation, markedly lower neck carriage, greatly reduced eye alertness, extreme lip separation, markedly increased base‐wide stance, increased occurrence and severity of crossed legs, buckled knees and⁄or fetlocks, pronounced loss of postural tone), the head level lower than the wither (at the carpal level)

### Analysis of samples

#### Chemicals

Water, acetonitrile, methanol and diethyl ether (HPLC‐grade) were obtained from Mallinckrodt Baker (Milan, Italy). Hydrochloric acid (37%) and sodium hydroxide (analytical reagent grade) were purchased from Carlo Erba (Milan‐Italy).

Since romifidine pure standard was not available, the registered and commercial preparation Sedivet^®^ (Boehringer Ingelheim Vetmedica, GmbH) was used as a reference standard, accepting the EU regulation that, concerning the veterinary medicinal products, establishes that the maximum acceptable deviation in the active substance content of the finished product shall not exceed ± 5% (Directive 2001/82/EC, 2001). Sedivet^®^ is an aqueous solution containing romifidine hydrochloride (10 mg/mL). A bottle of Sedivet^®^ was used to administer four horses the dosage of romifidine (100 *μ*g/kg), and to prepare the reference and the calibration curves to be used for the analysis of samples from the same four horses. A second bottle of Sedivet^®^, from the same batch of the first, was used to administer the additional four horses, and to prepare the reference and the calibration curves to be used for the analysis of samples from these last four horses.

#### HPLC instruments and chromatographic conditions

The HPLC system consisted of a Beckman System Gold equipped with a 507 autosampler fitted with a 50 *μ*L loop, coupled to an ultraviolet (UV)‐photodiode array Beckman 168 detector and GOLD release 4.0 software (Beckman Inst. INC). The UV absorption was measured at full spectrum (200‐300 nm) and at 215 nm. The analyses were carried out at room temperature on a Luna C18 column (particle size: 5 *μ*m, 250 × 4.6 mm, Phenomenex) protected with a guard column (Phenomenex SecurityGuard 4 × 2.0 mm). The mobile phase, consisting of a mixture methanol‐acetonitrile (2.5:1 v/v) and orthophosphoric acid (0.01 mol/L) (15:85 v/v), was at 0.7 mL/min flow rate. The injection volume was 50 *μ*L and the total run time was 30 min.

#### Stock and standard solutions

A stock of the standard solution (0.1 mg/mL) was prepared by diluting the commercial preparation of romifidine solution (10 mg/mL). The working solutions were prepared by further dilution of stock solution in H_3_PO_4_ 0.01 mol/L to analytical concentration in the range 0.02–2 *μ*g/mL. Both stock and reference standard solutions were stable for at least 3 months at 4 ± 2°C. Plasma and RBC calibration samples were prepared by pipetting suitable amounts of working solution of the analyte to 1 mL aliquots of blank pooled plasma or to 1 g aliquots of blank pooled RBC. Calibrators and QC (quality control) were prepared fresh for each batch and then treated exactly as horse's samples. Final concentrations of romifidine in the calibration standards were 0.01, 0.02, 0.05, 0.1, 0.2, 0.5, 1, 2 *μ*g/mL and *μ*g/g in plasma and RBCs respectively, separating the standard calibration for plasma and RBCs.

#### Sample preparation for plasma analysis

In a 10 mL borosilicate test tube, standard curve samples spiked with reference standard or unknown samples were acidified with 2.5 N hydrochloric acid (100 *μ*L; pH ≤ 3). Seven millilitres of diethyl ether were added, the tubes were capped and the mixture was gently stirred using a rotatory shaker for 10 min. After centrifugation (1200*g* for 5 min), the organic phase was discarded, and the aqueous phase was alkalinised (pH ≥ 12) by adding 100 *μ*L of 5 N sodium hydroxide. After addition of 7 mL of diethyl ether, the mixture was gently stirred using a rotatory shaker for 10 min. The samples were centrifuged (1200*g* for 5 min) and the organic layers were transferred into a new glass tube. This step was repeated twice and the second organic layer was transferred in the same glass tube containing the first one. The diethyl ether was evaporated under vacuum by Univapo (StepBio, Bologna, Italy). The residues were reconstituted with 250 *μ*L of H_3_PO_4_ 0.01 mol/L, and 50 *μ*L were injected into the HPLC system.

#### Sample preparation for RBC analysis

Since the high viscosity of cellular fraction containing RBCs, it was difficult to measure 1 ml using a pipette, therefore it has been decided to weigh accurately one gram by means analytical balance characterized by a precision of ±0.02 mg. One gram of RBCs was diluted with sodium dihydrogen phosphate buffer (0.1 mol/L, 5 mL) and homogenized using an Ultra‐Turrax T25 (Janke and Kunkel, Italy). Aliquots of the homogenate (2 mL) were transferred to conical glass screw‐capped tubes (15 mL), and 400 *μ*L of NaOH (5 N) were added to obtain a pH > 12. The tubes were briefly vortex‐mixed, and the samples were then extracted by shaking (10 min) with 8 mL of diethyl ether. After centrifugation (1200*g* for 5 min), the organic phase was transferred to a clean test tube (10 mL) containing 250 *μ*L of sulphuric acid (0.25 mol/L) and mixed for 10 min. The organic phase was decanted and discarded, and the aqueous layer was dried in a rotatory vacuum bench evaporator (40°C, UNIVAPO, StepBio, Bologna, Italy). The residue was dissolved using 200 *μ*L of H_3_PO_4_ (0.01 mol/L) and injected (50 *μ*L) into HPLC.

### *In vitro* study

In order to evaluate the concentration of romifidine in whole blood, in plasma and in red blood cells an *in vitro* assay was performed. Four tubes containing fresh heparinised horse whole blood (4.0 mL each) were pre‐incubated in a shaking water bath for 5 min at 37 ± 2°C, and spiked with stock solution to obtain a romifidine concentration of 1 *μ*g/mL. The reaction was terminated by transferring the tubes from the shaking bath to ice water bath at 2, 5, 10 and 30 min. At each time point aliquots of 1.0 mL were withdrawn from each tube and represent the whole blood fraction. The remaining whole blood (3 mL) was centrifuged (10 min; 1200*g*) and plasma was separated. One gram of corpuscular fraction containing RBCs was weighted and 1 mL of plasma measured and transferred in two new glass tubes. Whole blood, plasma and RBCs samples were kept at −20°C until analysis.

#### Method validation

Validation of the analytical procedure for the extraction and quantification of romifidine was carried out before analysis of the experimental samples. Drug‐free plasma samples (1 mL) or drug‐free cellular fraction containing RBCs (1 g) were fortified with romifidine standard reference solutions in order to prepare calibration curves ranging from 0.01 to 2 *μ*g/mL (or *μ*g/g for RBCs).

The linearity of the detector response was determined by the injection of spiked romifidine standards into blank matrices at different concentrations (0.01–2 *μ*g/mL, *μ*g/g for RBCs). The calibration curves were analysed using least square linear regression analysis, and the regression coefficient (*r* ^2^) was calculated.

The concentrations of romifidine in the unknown samples were calculated from their peak areas using the slope and the intercept of the calibration curves.

The recovery percentages were determined by comparing the peak areas obtained from the spiked plasma samples and spiked cellular fraction containing RBCs with the peak areas resulting from the reference standard solutions.

The inter‐day precision expressed as the coefficient of variation percentage (CV%) was checked by repeating (five times) the analysis of the spiked plasma (0.5 *μ*g/mL) and cellular fraction containing RBCs samples (0.5 *μ*g/g).

The limit of detection (LOD) was established by the Signal‐to‐Noise ratio at the romifidine retention time. The LOD was estimated taking into consideration a 1:3 ratio between the baseline noise and the concentration of the calibration standard. The quantification limit (LOQ) was the lowest concentration of each calibration curve, and the signal at the LOQ should be at least 10 times the noise level.

This study was carried out according to ISO 9001:2008 requirements.

### Statistical analysis

Plasma and RBC concentrations of romifidine were reported as mean ± standard deviation (SD). The Wilcoxon test was used to compare the concentrations in the two different compartments (plasma and RBCs), and the significance level was fixed at *P* < 0.05; statistical analysis was carried out using computer software (Med‐Calc 12.7.5, MedCalc Software, Acacialaan 22, B‐8400 Ostend, Belgium). The sedation scores (mean ± SD), plasma concentrations and RBC concentrations of romifidine were analysed using multilevel mixed‐effects linear regression analysis (Med‐Calc 12.7.5, MedCalc Software).

All pharmacokinetic parameters were reported as means ± SDs and were determined using computer software (WinNonlin 6.3; Pharsight Economy License, Pharsight Corporation, Mountain View, CA).The pharmacokinetic parameters evaluated were: A and B (the intercepts of the distribution and elimination phases), *α* and *β* (the rate constants of the distribution and elimination phases), *t*_1/2*α*_ and *t*_1/2*β*_ (the half lives of the distribution and elimination phases), AUC_0→∞_ (the area under the curve to infinity), Cl (body clearance), *V*d_ss_ (volume of distribution at a steady state), Vc (volume of distribution for the central compartment), *V*2 (volume of distribution for the shallow peripheral compartment), *C*_max_ (peak drug concentration), and MRT (mean residence time). *t*_1/2*β*_, was reported as harmonic mean. The individual plasma concentration‐versus‐time curves were fitted and the best compartment model was determined by application of the Akaike information criterion and Schwartz criterion (Schwartz 1978).

## Results

### Sedation

**Figure 1:**
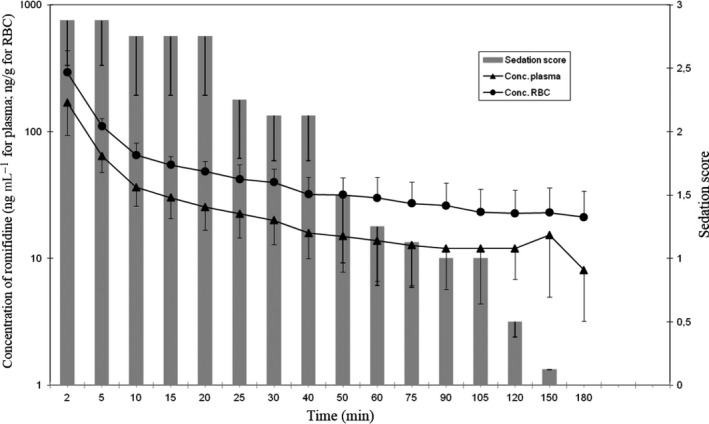
Plasma and red blood cell concentrations of romifidine and the sedation scores observed (average values) versus a time profile after intravenous administration.

Romifidine produced a good level of sedation in all animals as reported in Fig. 1. Sedation in all the horses was observable for up to 105 min (mean sedation score 1). Moreover, three horses at 120 min and one horse at 150 min were still sedated, and a sedation score of one was recorded. All the animals returned to normal without any behavioural changes at 180 min.

During clinical evaluation, the animals were in the corner of the stall and, in the first 40 min (mean sedation score 2.1 ± 0.35), they did not react when the operator entered the stall. The correlation between the sedation score with romifidine plasma RBC concentration highlighted a significant Spearman's coefficient (0.977 and 0.983 respectively) at each time point (*p* < 0.01) as show in Fig. 1.

### Chromatography

**Figure 2:**
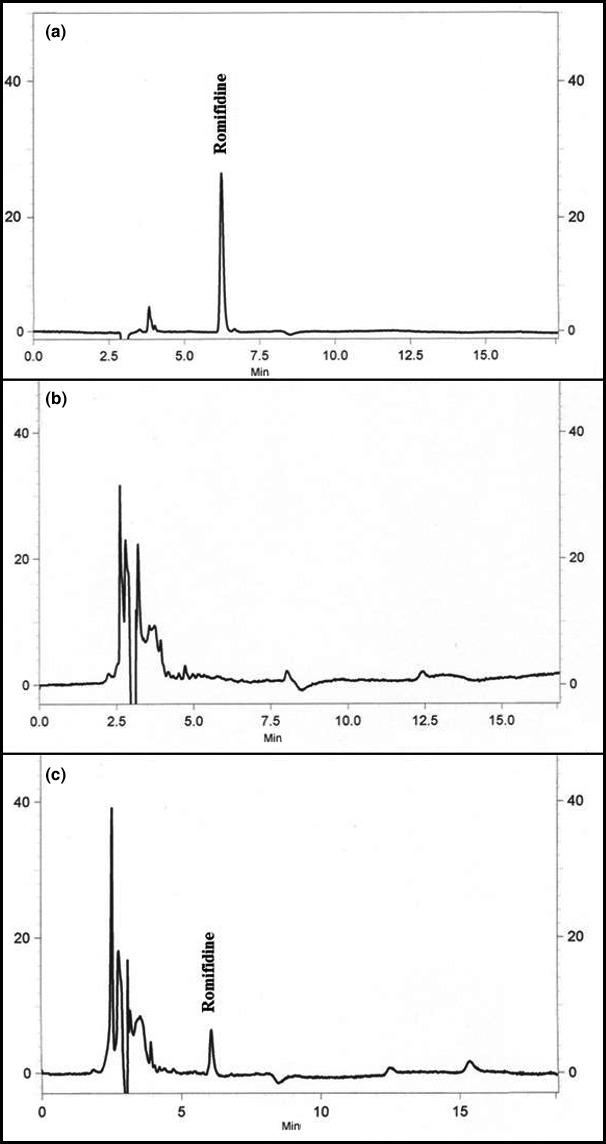
Chromatogram of 0.5 ng/mL standard of romifidine (a) and of blank RBCs (b), and a sample of RBCs collected after the administration of romifidine (c). RBC, red blood cell

Figure 2 shows a chromatogram of 0.5 ng/mL standard of romifidine (2‐a), of blank RBCs (2‐b) and a sample of RBCs collected after the administration of romifidine (2‐c). Under the chromatographic conditions adopted, the retention time was 6.5 min. The mean recovery percentages were 92 ± 3.1% for plasma and 84.6 ± 7.3% for RBCs. The CV% = 4.7 demostrated the high inter‐day precision.

The linear regression both in plasma and in RBCs in the range of 0.01‐2 *μ*g/mL or *μ*g/g respectively showed a high coefficient of correlation (generally exceeding 0.999 for the standard reference solutions and 0.99 for the calibration curves). The LOD and the LOQ were 0.001 *μ*g/mL and 0.01 *μ*g/mL respectively for plasma, and 0.001 *μ*g/g and 0.01 *μ*g/g for RBCs.

### Plasma and RBC concentrations

Romifidine was extracted from plasma and from the cellular fraction containing RBCs using liquid‐liquid extraction based on the method used for ketamine in our laboratory (Sori *et al*. 2013). The molecular structure of romifidine is different from that of ketamine. Both molecules are weakly basic and we verified that the alkalization allows the extraction with diethyl ether also for romifidine with a high recovery, as previously reported.

As shown in Fig. 1, the romifidine concentrations in the RBCs were significantly higher (*P* < 0.01) at all time points than those evaluated in the plasma. The results also showed that the release of romifidine from RBCs to plasma was consistent and continuous during the entire sedation period (Fig. 1).

**Figure 3:**
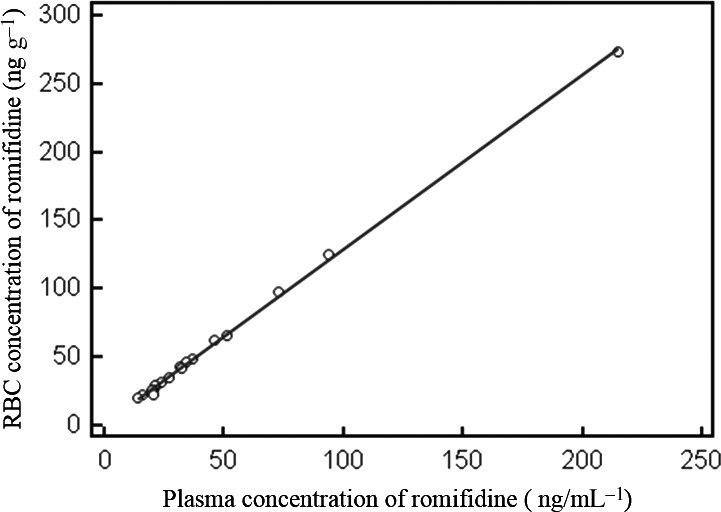
Representative linear relationship between romifidine concentrations in the plasma and red blood cells of all the horses (mean ± SD).

In Fig. 3, the diagram shows the fitted regression line between the concentrations of romifidine in plasma and its concentrations in RBCs for all the horses. The estimated regression equation was:

Y=2.2087+1.2732x

with a coefficient of determination (*R* ^2^) = 0.9990, an *F*‐ratio = 15844 and a significance level at *P* < 0.0001.

### Pharmacokinetic parameters

A bicompartmental model was determined to be the most appropriate for describing the romifidine concentration‐time profile for plasma. For all horses, the following equation was appropriate to describe the romifidine plasma concentrations:

$$C\left(t\right)=Ae^{-\alpha t}+Be^{-\beta t}$$

where C(*t*) is the plasma drug concentration at time *t*, e is the base of the natural logarithm, A is the *y*‐axis intercept, *α* is the slope for the distribution phase of the curve, B is the *y*‐axis intercept and *β* is the slope for the elimination phase of the curve.

The pharmacokinetic parameters of romifidine in plasma (means) are summarised in Table 2. As represented on a log‐linear axis (Fig. 1), the initial *α*‐phase of distribution decreased very rapidly (*α *= 0.22 ± 0.08/min) and was characterized by a short distribution half life (*t*_1/2*α*_=3.55 ± 1.36 min). The elimination phase (*β*‐phase) was characterized by a lower slope (*β = *0.006 ± 0.0004/min), indicating a slow decline in blood levels of romifidine as a result of the elimination processes (metabolism and excretion). This observation is supported by the t_1/2*β*_ and the body clearance values (148.67 ± 61.59 min and 22.55 ± 6.67 mL/min per kg respectively). Romifidine was still detected at the last time point of blood sampling (180 min) in each animal.

**Table 2:** Pharmacokinetics parameters (obtained from plasma concentration) of romifidine administered IV to horses (100 *μ*g/kg) as single bolus

Parameters	Units	Mean	SD
A	ng/mL	205.21	142.07
B	ng/mL	22.16	13.03
*α*	1/min	0.22	0.08
*β*	1/min	0.006	0.004
*t*_1/2*α*_	min	3.55	1.36
*t*_1/2 *β*_	min	119.28 (range 51.6–248.9)	
AUC_0→∞_	min ng/mL	4910.65	1963.68
CL	mL/min/kg	22.16	6.67
*V*d_ss_	mL/kg	3933.15	2002.46
*V*2	mL/kg	3319.53	1735.39
*C*_max_	ng/mL	227.37	151.53
MRT	min	176.75	75.93

A and B: intercepts of the distribution and elimination phases; *α* and *β*: the rate constants of distribution and elimination phases: *t*_1/2*α*_ and *t*_1/2*β*_: half lives of the distribution and elimination phases (*t*_1/2*β*_ is expressed as harmonic mean and range); AUC_0→∞_: the area under the curve to infinity; Cl: body clearance; *V*d_ss_: volume of distribution at the steady state; V2: volume of distribution for the shallow peripheral compartment; *C*_max_: peak drug concentration; MRT: mean residence time; SD, standard deviation.

### *In vitro* study

Despite differences between the horses, on the basis of *C*_max_ values of romifidine in plasma (227.37 ± 151.53 ng/mL) and in RBCs (575.47 ± 381.41 ng/mL), it can assume that RBCs might contain romifidine at about two times the plasma. Moreover, romifidine concentrates more in the RBC independently of the incubation time. The analysis of whole blood has confirmed this observation; indeed the concentrations in whole blood are similar to the average of the plasma and the RBC concentrations. Data concerning the whole blood concentrations were not reported because the extraction method applied to this matrix, despite having been verified on samples of known concentration, was not validated.

## Discussion

In this study, the pharmacokinetics of romifidine and the partitioning of the drug in the RBCs correlating the plasma and RBC concentration with the sedation score were evaluated. The initial hypothesis of romifidine distribution, and partitioning between plasma and RBCs after intravenous administration to horses was confirmed. In this study, after IV administration, the RBC concentrations of romifidine were approximately two time higher than those detected in plasma.

The maximal behavioural changes (sedation scores) were recorded between 2 and 40 min post injection, and the lowest detectable sedation score was recorded at 105 min. Our results differ from the data reported by Wojtasiak‐Wypart *et al*. (2012) where the authors administered a lower dose of romifidine (80 *μ*g/kg infused IV over 2 min) and behavioural changes were noted within 5 min of romifidine administration that became most prominent between 10 and 60 min and lasted for 120–150 min. In addition, residual sedation in all horses up to 120 min was recorded. The author supposed that these differences could be related to different dosage and different time of bolus infusion. However, Hamm *et al*. (1995) reported long sedation with a high detomidine dose (40 *μ*g/kg IV) as compared to 80 *μ*g/kg romifidine IV bolus. Freeman & England (2000) reported that using romifidine in horses at two different dosages (80–120 *μ*g/kg IV) reduced the horse response to auditory and tactile stimulation, and their degree of ataxia for up to 80 min.

The HPLC method used in this study is specific and sensitive for studying the disposition kinetics of romifidine in plasma and the concentration profile in RBCs. The plasma concentration vs. the time curve after IV romifidine administration in horses was well described by a bicompartmental model (Fig. 1). The same bicompartmental model has been chosen by other authors in pharmacokinetic studies regarding romifidine in horses (Hammer 2004; Wojtasiak‐Wypart *et al*. 2012).

Our results showed a rapid distribution phase (*t*_1/2*α*_ 3.55 ± 1.36 min) which was shorter than the value of romifidine obtained by Wojtasiak‐Wypart *et al*. (2012), followed by a long elimination half‐life (119.3‐range 51.6–248.9 min) similar to the data previously reported by Wojtasiak‐Wypart *et al*. (2012) and longer than the elimination half‐life described for detomidine and xylazine in horses (30 and 50 min respectively) (Garcia‐Villar *et al*. 1981; Grimsrud *et al*. 2009).

Our study show that, after IV administration, a large quantity of drug is detected into the red blood cells.

The plasma concentration vs. the time curve after IV romifidine administration in horses was described by a bicompartmental model (Fig. 1). The same bicompartmental model has been chosen by other authors in pharmacokinetic studies regarding romifidine in horses (Hammer 2004; Wojtasiak‐Wypart *et al*. 2012).

The rapid sequestration of romifidine into RBCs may also explain some significant pharmacodynamics effects observed in the study by Wojtasiak‐Wypart *et al*. (2012) such as the early and rapid decrease in heart rate and increase in arterial blood pressures, limited to only 5 min. The partitioning of drugs in the RBCs was first pointed out by Hinderling (1984, 1997). The RBCs represent the largest cell population of the blood. Previous studies have suggested that RBC partitioning depends, for the most part, on lipophilicity, molecular size and the chiral characteristics of the drug (Giebel & Passow 1960; Deuticke 1977). Romifidine has sufficient lipophilicity to pass through RBC membrane. If the blood vessels are considered as a “closed system”, then equilibrium should be reached when the ratio of unbound concentrations of the drug in the aqueous phase of the plasma and RBC cytosol remain constant as has previously been described for ketamine (Sori *et al*. 2013). On the contrary, in our study, the concentration of romifidine was higher in RBCs than in plasma at the same sampling time points in most horses and this could be explained by the parallel transport of the carrier‐mediated and the passive diffusion mechanisms. Moreover, romifidine, could bind to different RBC components, such as haemoglobin, protein, the plasma membrane and enzymes (Hinderling 1984, 1997).The RBC to plasma ratios were constant over time in the studied horses, independent of the sampling time point for blood collection. This finding indicates that RBCs do not act as a depot for romifidine in which the drug accumulates over time, as has been described for other drugs in human medicine, that is, for lometrexol (Synold *et al*. 1998). Therefore, it could be more likely a “weak and reversible” binding of romifidine with the membrane of red blood cells or with molecules inside the red blood cells.

The drugs in the red blood cells are inactive and can be released when the plasma concentrations of the drug decrease; this condition could allow maintaining the plasma concentration stable for a long period of time. In our experiment, six out of the eight horses had romifidine plasma concentrations higher than 10 ng/mL from 30 to 180 min. The minimum plasma concentration which produces sedation in horses is still not clear for all alpha_2_‐agonists but, in this study, a sedation score of one was recorded with a plasma concentration of romifidine higher than 10 ng/mL at 105 min.

In conclusion, the results showed that after a single bolus administration of romifidine, a partitioning in the RBCs was detected. This finding could explain the long sedative action of romifidine, observed by different authors in horses after a single bolus injection.

Additional *in vitro* study should be carried out to improve knowledge regarding the mechanism of the partitioning of romifidine in RBCs. Additional pharmacokinetic and pharmacodynamic studies of romifidine should also be conducted in anaemic and polycythemic horses.

## Source of funding

This research did not receive any specific grant from funding agencies in the public, commercial, or not‐for‐profit sectors.

## Conflict of interest

The authors declare that there is no conflict of interest regarding the publication of this manuscript.

## Ethical statement

The study was approved by the Scientific Ethics Committee for Experimentation on Animals of Alma Mater Studiorum‐University of Bologna. The care and handling of the animals were in accordance with the provisions of European Economic Community Council Directive 86/609, adopted by the Italian Government (D.L. 27/01/1992 no. 116).
